# Immunolocalization of Keratan Sulfate in Rat Spinal Tissues Using the Keratanase Generated BKS-1(+) Neoepitope: Correlation of Expression Patterns with the Class II SLRPs, Lumican and Keratocan

**DOI:** 10.3390/cells9040826

**Published:** 2020-03-30

**Authors:** Anthony J. Hayes, James Melrose

**Affiliations:** 1 Bioimaging Research Hub, Cardiff School of Biosciences, Cardiff University, Cardiff CF10 3AX, Wales, UK; HayesAJ@cardiff.ac.uk; 2 Graduate School of Biomedical Engineering, University of New South Wales, Sydney, NSW 2052, Australia; 3 Raymond Purves Laboratory, Institute of Bone and Joint Research, Kolling Institute of Medical Research, Northern Sydney Local Health District, Faculty of Medicine and Health, University of Sydney Royal North Shore Hospital, St. Leonards, NSW 2065, Australia; 4 Sydney Medical School, Northern, University of Sydney at Royal North Shore Hospital, St. Leonards, NSW 2065, Australia

**Keywords:** keratan sulfate, keratocan, lumican, intervertebral disc, spinal cord, endochondral ossification, neural development

## Abstract

This study has identified keratan sulfate in fetal and adult rat spinal cord and vertebral connective tissues using the antibody BKS-1(+) which recognizes a reducing terminal N-acetyl glucosamine-6-sulfate neo-epitope exposed by keratanase-I digestion. Labeling patterns were correlated with those of lumican and keratocan using core protein antibodies to these small leucine rich proteoglycan species. BKS-1(+) was not immunolocalized in fetal spinal cord but was apparent in adult cord and was also prominently immunolocalized to the nucleus pulposus and inner annulus fibrosus of the intervertebral disc. Interestingly, BKS-1(+) was also strongly associated with vertebral body ossification centers of the fetal spine. Immunolocalization of lumican and keratocan was faint within the vertebral body rudiments of the fetus and did not correlate with the BKS-1(+) localization indicating that this reactivity was due to another KS-proteoglycan, possibly osteoadherin (osteomodulin) which has known roles in endochondral ossification. Western blotting of adult rat spinal cord and intervertebral discs to identify proteoglycan core protein species decorated with the BKS-1(+) motif confirmed the identity of 37 and 51 kDa BKS-1(+) positive core protein species. Lumican and keratocan contain low sulfation KS-I glycoforms which have neuroregulatory and matrix organizational properties through their growth factor and morphogen interactive profiles and ability to influence neural cell migration. Furthermore, KS has interactive capability with a diverse range of neuroregulatory proteins that promote neural proliferation and direct neural pathway development, illustrating key roles for keratocan and lumican in spinal cord development.

## 1. Introduction

**Figure 1:**
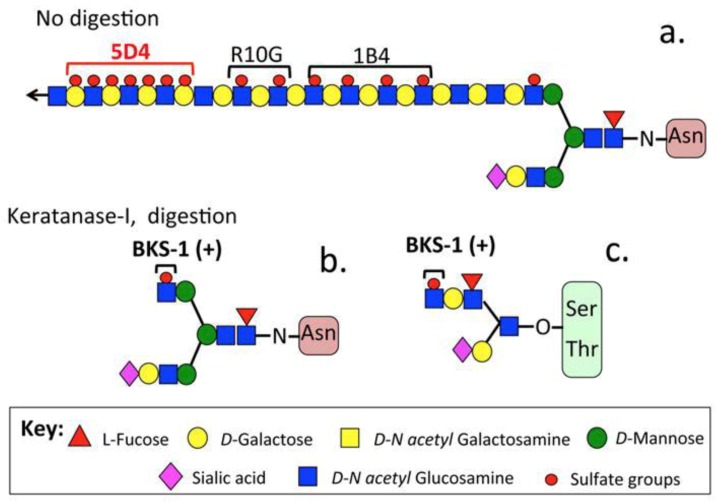
Structure of a typical KS-I chain and identification of high charge density 5-D-4 and low charge density R10G and 1-B-4 antibody binding sites (**a**) in N- (**b**) and O-linked KS chains (**c**).

Keratan sulfate (KS) is a widely distributed glycosaminoglycan (GAG) component of proteoglycans (PGs) in the extracellular matrix (ECM) and on cell surfaces of tensional and weight-bearing connective tissues such as ligaments, tendons, articular cartilage, and intervertebral discs (IVD), in addition to corneas and the central and peripheral nervous systems (CNS/PNS) [1,2,3]. Historically, most of the information on the spatio-temporal distribution and function of KS-PGs has been gleaned through the use of the monoclonal antibody (MAb) 5-D-4 which detects disulfated heptasaccharide regions of high charge density in KS chains [4]. KS is initially synthesized as a polylactosamine chain sulfated on its GlcNAc residues in a monosulfated KS glycoform [5], and with tissue maturation galactose residues of the GlcNAc-Gal KS repeat disaccharide become progressively sulfated at C6 leading to regions of disulfation on the KS chains. KS is heterogeneous and contains variably distributed regions which are non-sulfated, and mono- and disulfated [1]. While heptasaccharide groupings of disulfated regions on KS are detected by MAb 5-D-4, this antibody provides no information on mono-or non-sulfated regions of KS [6]. As MAb 5-D-4 is incapable of detecting low charge density KS species, its application to embryonic and fetal connective tissues may under represent the quantity and distribution of KS GAGs which have yet to develop appreciable levels of highly sulfated KS epitopes. Antibodies to the mono-sulfated (MAb R10-G, 1B4) [7] and non-sulfated polylactosamine regions of KS (MAb ‘i’) [8] are now available and, in combination with specific KS depolymerizing enzymes, functional roles for these regions are now emerging [9]. BKS-1 immunolocalization is therefore a more accurate quantitative measure of KS since one BKS-1 epitope is labeled per KS chain in all KS glycoforms irrespective of their charge status (Figure 1).

MAb BKS-1 identifies KS in early tissue development in KS-PGs containing low sulfation KS glycoforms as well as highly sulfated KS in mature tissues. In corneal tissues, MAb 5-D-4 and BKS-1 detect similar corneal KS-PG populations by Western blotting [10] and, in immunolocalizations, BKS-1 displays a more subtle localization of KS than 5-D-4 [11], possibly due to the stoichiometry of 5-D-4 and BKS-1 antibody binding to KS chains. BKS-1 staining is quantitative, thus the relative levels of KS in different regions of a given tissue can be precisely compared. Furthermore, whereas MAb 5-D-4 detects longer, more highly sulfated KS chains, BKS-1 detects shorter, less highly sulfated KS chains, typical of those found in lumican, keratocan, and proline/arginine-rich end leucine-rich repeat protein (PRELP) in fetal or embryonic tissues. The presence of these low sulfation KS glycoforms has previously been under-appreciated [1]. For example, a mono-sulfated KS glycoform in electro-sensory tissues of the Ampullae of Lorenzini in elasmobranch sharks and rays is the sole GAG found in these tissues [12] and is the most sensitive proton detection agent known in nature [13]. A natural killer (NK) cell-restricted KS-glycoprotein (PEN5), a developmentally-regulated post-translational modification of the platelet selectin glycoprotein ligand-1 (PSGL-1), is a unique binding site for L-selectin expressed by platelets, endothelial cells, and leukocytes serving as a functional NK-homing-trafficking receptor delivery system and a molecular switch in the activation of cytolytic NK cells in the innate immune response [14]. KS is also a functional cell surface component of the glycocalyx in human embryonic stem cells with specific roles in their differentiation into defined cell lineages [15].

A recent study with corneal KS also showed that this GAG interacted with a diverse range of morphogens, growth factors, nerve growth factor receptors, and members of the Robo, Slit, Ephrin, Ephrin receptor, and Semaphorin families of neuroregulatory proteins [16]. The strong localization of BKS-1(+) KS observed in the spinal cord (SC) of adult rats in the present study is consistent with roles for KS in neuroregulation and neural guidance in the CNS/PNS [3] and provides further evidence that low sulfation KS glycoforms may have novel functional roles in the growth and development of the SC and vertebral column.

## 2. Materials and Methods

All materials and supplier details were as specified earlier [17].

### 2.1. Tissues

Human cartilage was donated by total knee replacement patients using the North Sydney Area Health Authority Ethics protocol 0404-103M (Oct 2004–2008): J Melrose, C Little, D Sonnabend—“Pathobiology of the small leucine rich repeat proteoglycans in cartilage, intervertebral disc and tendon degeneration” which covers the use of human tissues discarded at surgery. Late fetal (embryonic days 19–20) and adult (4 month) spinal tissues from white Wistar rats were used in this study as described previously [18]. These were killed humanely using a Schedule 1 procedure (Animals Scientific Procedures Act, 1986, UK).

### 2.2. Antibodies

The LUM-1 and KER-1 core protein antibodies used in this study (Table 1) have been described previously [17,18] as has the BKS-1(+) KS stub neoepitope antibody [11,19] (Figure 1). Please note that the KS antibodies used in the present study detect sulfated epitopes in the KS and should not be confused with anti-aminoacyl-tRNA synthetase antibodies which have also been referred to as KS antibodies [20] or the anti-cyclin D1/D2 5-D-4 antibody [21].

The LUM-1 and KER-1 monoclonal antibodies used in this study were kind gifts from Dr Briedgeen Kerr prepared as part of her PhD studies, Keratan Sulfate Metabolism in Connective Tissue Proteoglycans conducted at The University of Cardiff, UK (2005). The authenticity of these antibodies was determined using extracts of human knee articular cartilage which contains lumican and keratocan [17]. Human recombinant lumican and keratocan proteins produced in *Escherichia coli* and HEK 293T cells were purchased from OriGene (OriGene Technologies, Inc., Rockville, MD 2085, USA) and used as positive control standards in Western blots. A rabbit polyclonal antibody to keratocan (KTN, cat # ab113115) raised to C-terminal amino acids 227-257 was also obtained from abcam (Cambridge, UK). A rabbit monoclonal Lumican antibody (B9, cat # sc-166871) was obtained from Santa Cruz Biotechnology (Texas, USA). A rabbit polyclonal lumican antibody (PR-353) to the C-terminal amino acid sequence LRVANEVTLN was a gift from Prof Peter Roughley, McGill University, Canada. The KTN, B-9, and PR-353 antibodies were used in Western blotting with human knee cartilage extract or recombinant lumican and keratocan proteins to confirm that KER-1 and LUM-1 identified keratocan and lumican core proteins as shown previously in a number of studies [5,17,22,23,24,25].

**Table 1:** Keratocan and lumican antibodies used in this study.

Antibody, Ab Class (Immunizing Antigen Used)	Source	Specificity	Reference
LUM-1, Mouse IgG monoclonal (lumican core protein)	Bridgeen Kerr, Cardiff University, UK	Mouse monoclonal IgG to 51 kDa core protein	[26]
KER-1, Mouse IgG monoclonal (keratocan core protein)	Bridgeen Kerr, Cardiff University, UK	Mouse monoclonal IgG to 38 kDa core protein	[26]
PR 353, Rabbit polyclonal (lumican C-terminal peptide LRVANEVTLN)	Peter Roughley, McGill University, Canada	Rabbit polyclonal Ab identifies LRVANEVTLN C-terminal peptide in lumican species	[17]
KTN, Rabbit polyclonal Ab, (C-terminal amino acids 227–257 of keratocan)	Abcam (cat # ab113115), UK	Rabbit polyclonal Ab to 38 kDa keratocan core protein	[27]
B9 Mouse monoclonal IgG (lumican core protein)	Santa Cruz (cat # sc-166871), USA	Rabbit monoclonal to 51 kDa clumican ore protein	[28,29]
BKS-1(+) Mouse monoclonal IgG (keratanase digested KS)	Bridgeen Kerr, Cardiff University, UK	Galactosamine-6-sulfate-galactose disaccharide in KS linkage region	[26]

### 2.3. Histology

Tissues were fixed in 10% *w*/*v* neutral buffered formol saline, decalcified in 2% *v*/*v* nitric acid until radiologically clear, and then processed into paraffin wax using standard histological methods. Serial sections were cut through the paraffin blocks containing embedded vertebral body/intervertebral disc segments in the sagittal plane and 6 μm sections were collected onto Histobond glass histology slides (R.A. Lamb, UK/Thermo Fisher, www.thermofisher.com).

### 2.4. Immunohistochemistry

Tissue sections were de-waxed then rehydrated in sequential washes of xylene and graded ethanol to water and digested with a mixture of chondroitinase ABC (0.025 U/mL) and keratanase-I (0.4 U/mL) in 50 mM Tris–HCl pH 6.5 0.1 M NaCl for 3 h at 37 °C before washing in phosphate buffered saline (PBS) pH 7.4. Endogenous peroxidase was initially blocked by incubation of the sections in 0.3% *v*/*v* hydrogen peroxide in water for 1 h. After washing, non-specific protein binding in the sections was blocked with normal horse-serum for 30 min. Primary antibodies KER-1, LUM-1, and BKS-1 were used at a 1:20 dilution [11,19] and incubated overnight with tissue sections at 4 °C. Controls sections were incubated with irrelevant naïve immunoglobulins of the same isotype or the primary antibody was omitted, being replaced with PBS, pH 7.4. All staining with immunoglobulin control samples were negative, showing no non-specific antibody labeling. Primary antibody localizations were visualized using the Vector ABC universal immunoperoxidase labeling kit (Vector Laboratories, Burlingame, CA, USA) using NovaRed peroxidase substrate (Vector Laboratories). The sections were then washed, counterstained with haematoxylin, and mounted under coverslips with DPX mountant and imaged under brightfield optics using a Surveyor slide scanning system (Objective Imaging, Cambridge, UK) equipped with a QICAM Fast 1394 color CCD digital camera (Teledyne QImaging, Canada). BKS-1(+) sections required keratanase digestion to generate the BKS-1(+) epitope. Omission of the keratanase digestion step resulted in an absence of generation of the BKS-1(+) epitope.

Rat tissue sections were also stained with 0.05% alcian blue 8GX to visualize sulfated GAG, or immunolabeled with antibodies to type II and VI collagens (1 μg/mL) overnight at 4 °C as specified earlier [30] to provide tissue context within the spinal tissues.

### 2.5. Extraction of PGs from Rat and Human Tissues

Finely diced tissue from 12 rat IVDs from two 4 month old male Wister rats and SC from 2 rats were extracted in 10 volumes of 4 M GuHCl 0.5 M sodium acetate (pH 5.8) containing 10 mmol/L EDTA, 20 mmol/L benzamidine, and 50 mmol/L 6-aminohexanoic acid for 48 h at 4 °C. PGs were also extracted from surgically discarded total knee replacement human knee articular cartilage using 4 M GuHCl for antibody validation studies.

### 2.6. Chondroitinase ABC and Keratanase-I Digestion of Proteoglycan Samples

Freeze dried tissue extracts were re-dissolved (2 mg dry weight/mL) in 0.1 M Tris 0.03 M acetate buffer (pH 6.5), and aliquots (0.5 mL) were pre-digested with chondroitinase ABC (0.1 U) and keratanase-I (0.05 U) overnight at 37 °C.

### 2.7. Lithium Dodecyl Sulfate PAGE and Western Blotting

Chondroitinase ABC and keratanase-I digested PG samples (0.1 mL; 2 mg/mL) were mixed with 4 × lithium dodecyl sulfate PAGE application buffer (35 μL) and 500 mM dithiothreitol (15 μL) heated at 70 °C for 30 min, cooled, and 25 μL aliquots electrophoresed under reducing conditions on 10% NuPAGE Bis-Tris gels at 200 V constant voltage for 50 min using NuPAGE MOPS (3- [N morpholino]-propanesulfonic acid) pH 7.0 SDS electrophoresis buffer. Gels were electroblotted to nitrocellulose membranes (0.22 μm) in NuPAGE transfer buffer containing 10% methanol at 30 V constant voltage for 1 h. SeeBlue-2 pre-stained protein molecular weight standards (InVitrogen Australia, Mount Waverley, Vic, Australia) were also electrophoresed. Blots were blocked for 3 h in 5% bovine serum albumin in 50 mM Tris–HCl 0.15 M NaCl (pH 7.2; TBS) and anti-lumican, anti-keratocan, or anti-BKS-1 antibodies (1 μg/mL) added overnight in 2% bovine serum albumin in TBS. Goat anti-rabbit or anti-mouse IgG alkaline phosphatase conjugated secondary antibodies (1/5000 dilution) were added for 1 h. The blots were then washed in TBS and developed with NBT/BCIP (nitro-blue tetrazolium chloride/5-bromo-4-chloro-3’-indolyphosphate) substrates in 0.1 M Tris–HCl pH 9.5 containing 5 mM MgCl_2_ for 20 min at room temperature.

## 3. Results

The schematic shown in Figure 1 summarizes the structural epitopes within the KS GAG chain structure that are identified by some of the most commonly used KS antibodies. BKS-1 detects a reducing terminal N-acetyl glucosamine-6-sulfate neo-epitope exposed by keratanase-I digestion, i.e., BKS1(+). The (+) designation is used to signify that keratanase-I is required to expose this epitope.

### 3.1. Western Blotting

**Figure 2:**
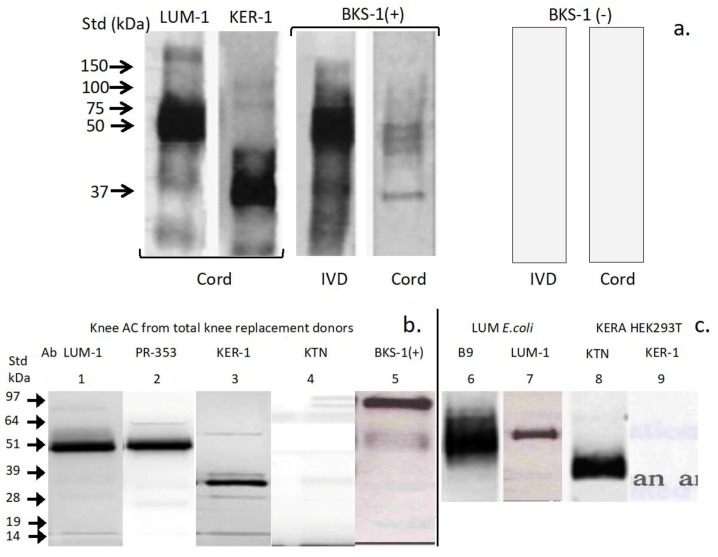
Western blotting validation of the specificity of the KER-1 and LUM-1 antibodies and comparison of the KS species identified by BKS-1 with keratanase pre-digestion (+) or without keratanase digestion (-) in extracts of 4 month rat spinal tissues (**a**), human articular cartilage, (**b**) and positive identification of recombinant lumican (LUM *E. coli*) and keratocan (KERA HEK 293T) standard proteins (**c**). The PG species detected in extracts of human articular cartilage (AC) by a number of commercial and gifted lumican (PR-353; B9, Santa Cruz cat # sc-166871) and keratocan (KTN, abcam cat # ab113115) were also compared with those identified by LUM-1 and KER-1 (**b**). All samples were predigested with keratanase-I.

**Figure 3:**
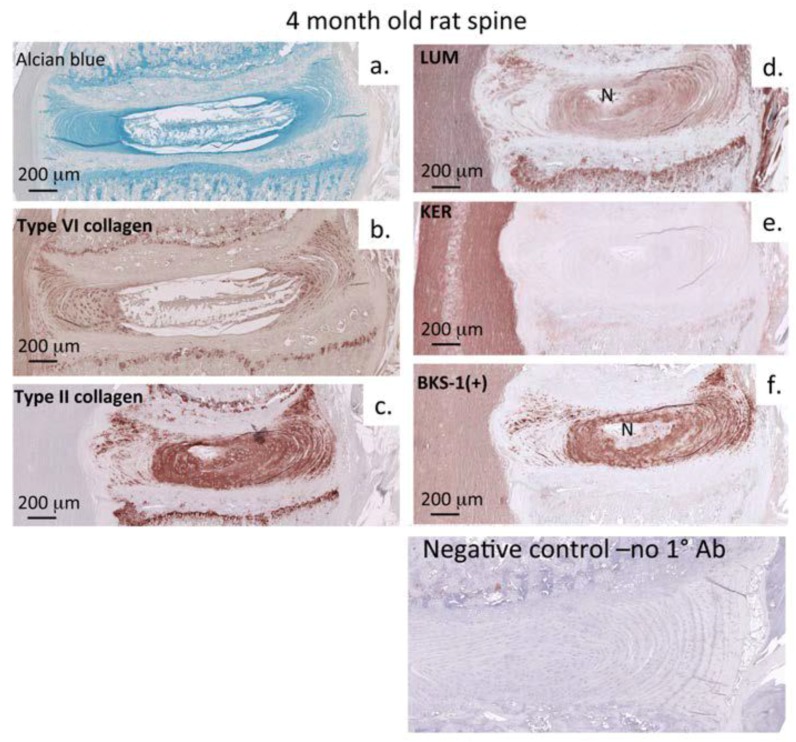
Visualization of the distribution of rat spinal components. Histochemistry/immunohistochemistry of alcian blue proteoglycan (**a**), type VI collagen (**b**), type II collagen (**c**), lumican (**d**), keratocan (**e**), and BKS-I (+) (**f**) in a 4 month old rat spinal segment. Alcian blue and antibodies towards collagen types II and VI have been used to delineate the constituent spinal connective tissues. Lumican is immunolocalized to the nucleus pulposus and inner annulus fibrosus and prominently in the vertebral growth plate cartilages and anterior longitudinal ligament, however keratocan is only weakly immunolocalized to the vertebral growth plate. BKS-1(+) epitope is prominently immunolocalized in the nucleus pulposus, inner annulus fibrosus, and the vertebral growth plates.

Western blotting of extracts of SC and IVD from adult (4 month) old rat spinal tissues showed that both tissues contained lumican and keratocan and BKS-1(+) positive KS-PGs (Figure 2a). Lumican and keratocan core proteins also contained the BKS-1(+) epitope (Figure 2a). The LUM-1 and KER-1 antibodies displayed similar KS-PG detection profiles to those detected by the B-9 and PR-353 anti-lumican and KTN anti-keratocan antibodies in human articular cartilage tissue extracts (Figure 3b) and to recombinant 51 kDa lumican and 37 kDa keratocan core protein standards (Figure 3c). The BKS-1(+) positive lumican and keratocan PG core protein species detected in the present study demonstrate that these PGs contain low sulfation KS glycoforms in embryonic and neonatal spinal cord while highly charged 5-D-4 positive KS chains are also known to be present on these PGs in mature and in pathological connective tissues.

### 3.2. Immunohistochemistry: Adult Rat Spinal Tissue

**Figure 4:**
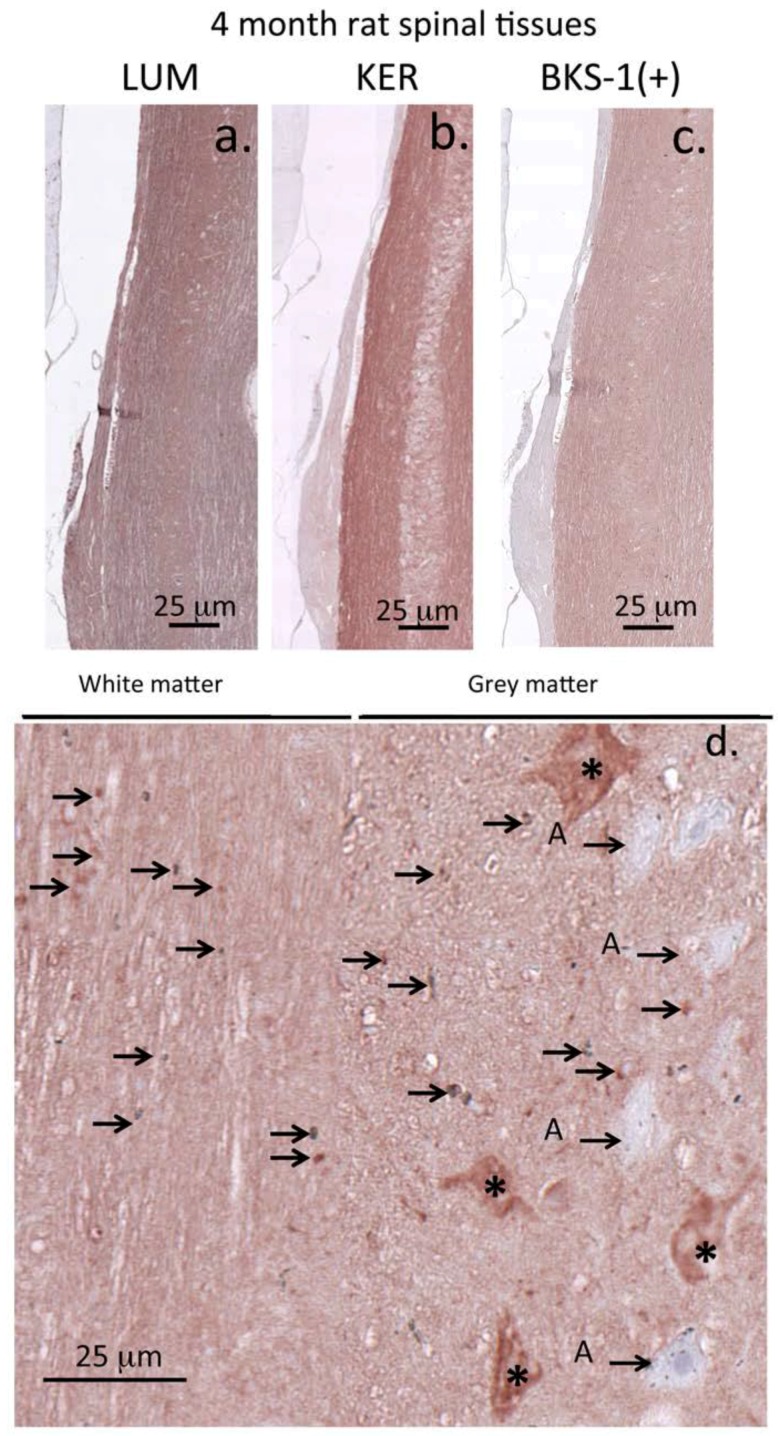
Immunolocalization of keratocan (**a**), lumican (**b**), and BKS-1 (+) KS neo-epitope (**c**) in 4 month old rat spinal tissue shows them to be widely distributed throughout the spinal cord. A higher power image of BKS-1(+) shows reactivity prominently localized throughout the spinal cord extracellular matrix and also labeled glial cells and neurons. (**d**) Glial cells are arrowed and motor neurons labeled with an asterisk, astrocytes (A) are labeled with a small arrow. Scale bars 100 μm. Chromogen NovaRED.

**Figure 5:**
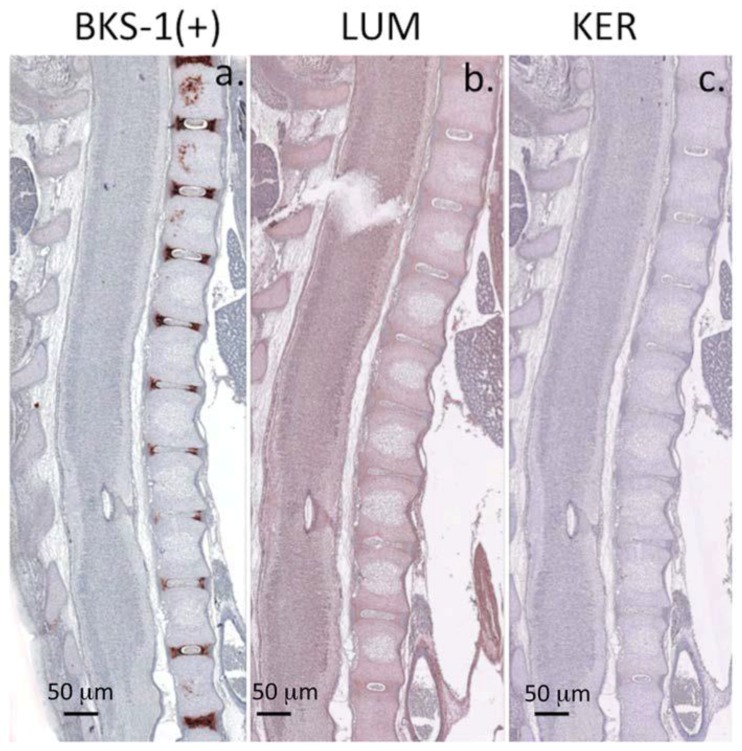
Macroscopic longitudinal sections depicting the immunolocalization of BKS-1(+) KS neoepitope in chondroitinase ABC, keratanase I-digested spinal tissue sections (**a**), lumican (**b**), and Keratocan (**c**). BKS-1(+) reactivity is evident in the pre-ossification centers in the vertebral bodies, and the cartilaginous inner annulus fibrosus of the developing intervertebral discs (**a**). BKS-1 (+) is also immunolocalized to the notochordal sheath surrounding notochordal remnants in the central intervertebral disc at some levels but not in the notochord itself which has been lost by this stage. Staining for BKS-1 (+) epitope is negative in the spinal cord at this stage of spinal development and only weak for lumican. Scale bars 100 μm. NovaRED chromogen.

At 4 months the rat vertebral column was skeletally mature and its associated connective tissues had fully formed. Alcian blue staining for sulfated GAG (Figure 2a) and IHC for collagen types II and VI (Figure 3b,c) were used to delineate the constituent musculoskeletal tissues, highlighting the positions of the vertebral growth plates (GP), the cartilage end plates (CEP), the NP and AF of the IVD, and adjacent SC tissue. Immunohistochemistry of adult (4 month) rat spinal tissues showed that BKS-1(+), lumican, and keratocan were all identifiable within the SC ECM, IVD, and GP (Figure 3d–f and Figure 4a–c). Lumican was prominently immunolocalized to the GP using the LUM-1 core protein Ab (Figure 3d) but not with BKS-1(+) (Figure 3f) suggesting that the form of lumican in the GP may be unsulfated; further studies need to be undertaken to confirm this observation. Higher magnifications of the SC tissue showed that BKS-1(+) was broadly localized throughout the ECM and associated with glial cells and neurons, but not astrocytes (Figure 4d). This contrasted with the fetal rat SC tissue in which the BKS-1(+) epitope could not be detected (Figure 5a).

### 3.3. Immunohistochemistry: Fetal Rat Spinal Tissue

**Figure 6:**
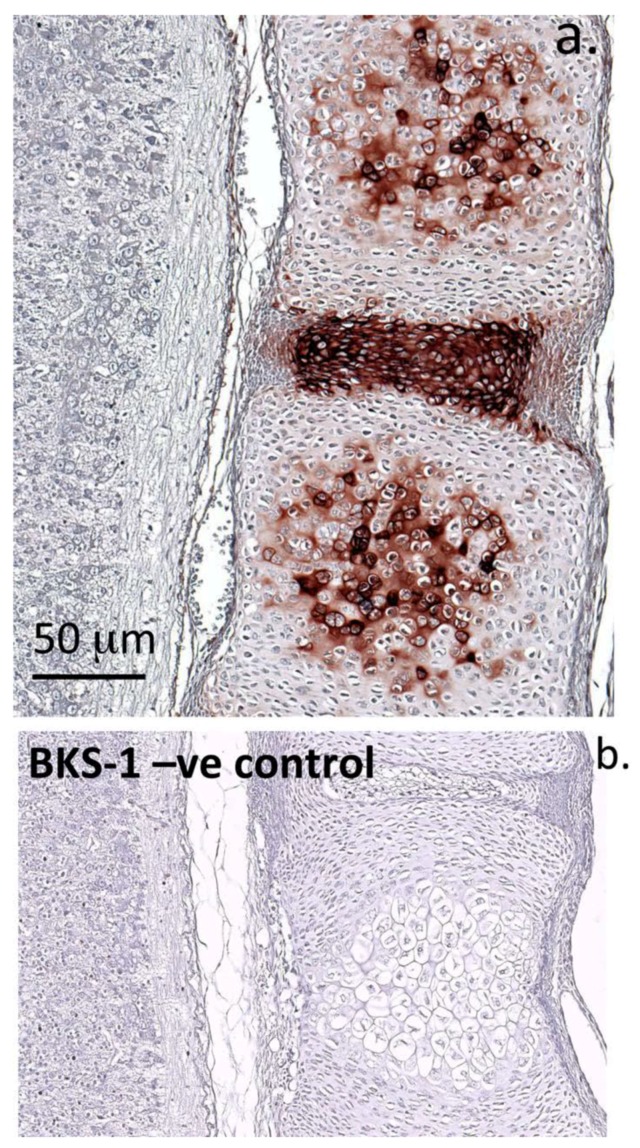
Immunolocalization of BKS-I(+) (**a**) in a late fetal rat spinal segment showing its localization in the pre-ossification centre within the vertebral body rudiment, and the cartilaginous inner annulus fibrosus of the disc. Note that the nucleus pulposus is outside the section plane of this parasagittal tissue slice taken through the disc. Chromogen used was novaRED. A negative control image is presented in (**b**).

Immunolocalization studies of late fetal rat spinal tissues with BKS-1(+) showed strong ECM labeling of keratanase-generated KS neoepitopes within the cartilaginous inner AF of the developing IVD (Figure 5a). Labeling was also present within the notochordal sheath surrounding the NP but was absent from the notochordal remnant tissue within the nascent NP. Strong labeling was also noted within the pre-ossification centers of the vertebral rudiments (particularly prominent rostrally in the section plane shown). However, there was a conspicuous absence of BKS-1(+) labeling within the fetal SC tissue (Figure 5a and Figure 6a). In contrast, lumican (Figure 5b) was weakly detected within the fetal SC and vertebral cartilages, but was absent from their pre-ossification centers. Keratocan labeling (Figure 5c) was more restricted in its distribution and associated with only small numbers of cells within the developing SC and transitional tissue of the IVD. It was also absent from the vertebral pre-ossification centers; however, it was prominent within the stratum basale of the dermis (data not shown).

Labeling of BKS-1(+) was highly prominent within the NP and AF of the IVD; however, it was not detected in the CEPs or adjacent GPs (Figure 6a). Lumican had a similar distribution to BKS-1(+), occurring within the NP and AF. It too was absent from the CEPs; however, unlike BKS-1(+) it was prominent in the vertebral GPs and the posterior longitudinal ligament (Figure 6a). Keratocan, whilst immunolocalizing strongly with the SC tissue was conspicuously absent from the adjacent IVD tissue and thus served as a negative control for the BKS-1 localizations (Figure 6b)

**Figure 7:**
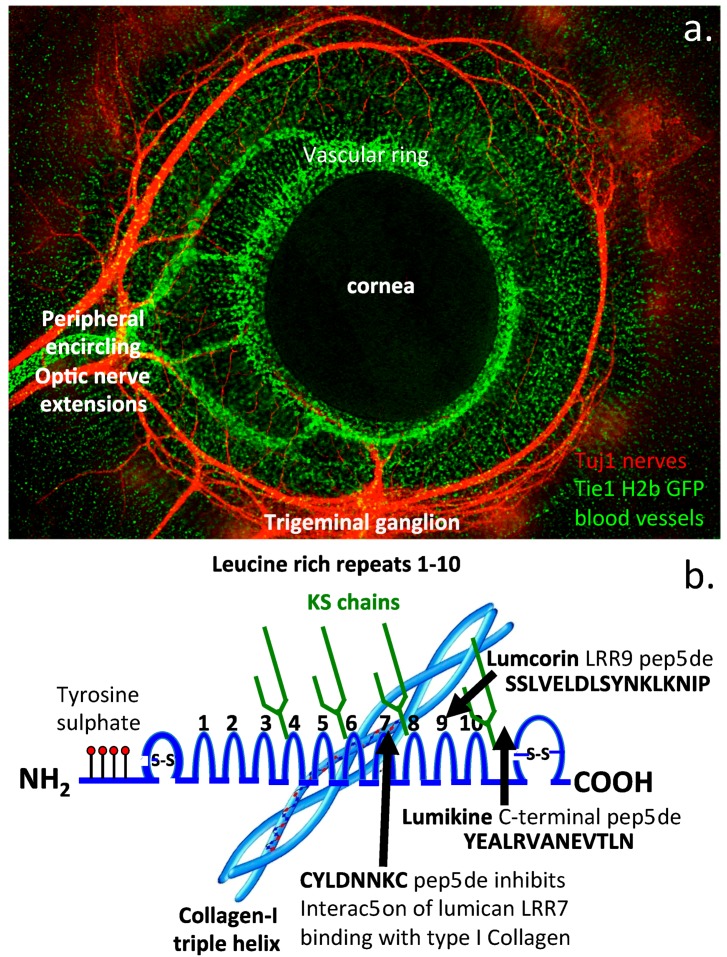
Composite figure depicting the distribution of nerves and blood vessels around the cornea in an embryonic quail and illustration of the structural organization of lumican which contributes to its functional properties in tissues. A fluorescent image depicts the developmental peripheral trigeminal nerve and associated blood vessels regulated by keratocan and lumican in the E7 quail eye. The trigeminal nerve was visualized using a Tuj-1 antibody. Ocular blood vessels were visualized using green fluorescent protein labeled TG–Tie-1H2b under the direction of the endothelial orphan receptor Tie-1 and plasminogen receptor histone 2B promoters. Image kindly supplied by Associate Prof Peter Ligwale, Rice University, USA (**a**). Diagrammatic depiction of lumicans structure and the leucine rich repeat 7 mediated interaction of lumican with type I collagen which regulates collagen fibrillogenesis (**b**). The synthetic peptide **CYLDNNKC** inhibits this interaction between lumican LRR7 and type-I collagen. A peptide, lumcorin from LRR9 also has α2β1 integrin binding and cell regulatory properties. Lumikine a C-terminal lumican peptide also binds ALK5/TGFβR1 and has wound healing properties.

Keratocan and lumican both have documented roles in the regulation of nerve migration during the development of the CNS/PNS. Figure 7 shows the distribution of blood vessels and encircling nerves in the E7 quail eye in regions known to be regulated by keratocan (Figure 7a). Lumican also regulates endothelial cell migration and blood vessel development and the formation of regular orthogonally arranged fine collagen fibers in the cornea essential for optimal clarity. Thus keratocan and lumican both have important roles in the development of the quail eye and ECM components in general. A diagrammatic representation of lumican and its structural organization important for its functional properties is shown (Figure 7b), and central leucine rich repeats (LRRs) and lumicans interactive regions with type I collagen are depicted. The lumcorin LRR9 and C-terminal lumikine ALK5/TGFBR1 binding peptide are also shown (Figure 7b).

## 4. Discussion

BKS-1(+) members of the small leucine rich proteoglycan (SLRP) family, including lumican and keratocan, have previously been reported in the development of the annulus fibrosus of the rat IVD [18]. In the present study we report on their wider distribution in rat spinal development using IHC and SDS-PAGE and Western blotting. We show that BKS-1(+), lumican, and keratocan have interesting spatio-temporal tissue-specific distributions that suggest they play complex roles in the development of the spinal cord and many of the constituent musculoskeletal connective tissues of the vertebral column. The BKS-1(+) reactivity observed within the pre-ossification centers of the vertebral rudiments during fetal development of the spine indicates the involvement of a KS substituted PG other than keratocan and lumican in their early development as there was no correlation in the labeling patterns of BKS-1(+) with these SLRPs. The identity of this PG is unknown; however, osteoadherin is a strong candidate as this KS SLRP is localized in bone tissue and upregulated during endochondral ossification and dentin formation [31,32,33,34,35,36]. Osteoadherin (osteomodulin) is a 49,116-Da protein containing 11 leucine-rich repeats (LRRs), 3-4 tyrosine sulfate residues at the N-terminus, and six potential glycosylation sites for N-linked KS chains within the LRR region. Osteoadherin shows 42% sequence homology to keratocan and 37–38% identity to fibromodulin, lumican, and PRELP [34]. Osteoadherin promotes α_v_β_3_- integrin mediated cell binding and has been isolated as a minor, leucine and aspartic acid-rich KS-PG from mineralized tissues [36]. Osteoadherin is a relatively acidic protein which binds to hydroxyapatite and to osteoblasts through α_v_β_3_- integrin and has been immunolocalized to pre-dentin during tooth formation [37].

The present study indicated that lumican and keratocan were prominent matrix components of the adult rat SC. These SLRPs were originally identified as bone and cartilage matrix PGs that bind and regulate growth factors such as TGF-β1 [38] to modulate collagen fibrillogenesis. However, lumican and keratocan may also regulate SC cell populations and collagen fibrillogenesis in the developmental SC [39]. Lumican was also prominently detected as a component of the vertebral growth plate using the LUM-1 core protein antibody but was not detected using the BKS-1 MAb thus it may be present as a non-glycanated form in the growth plate. Non-glycanated forms of decorin and biglycan have previously been identified in the IVD [40,41], articular cartilage [42], and human nasal cartilage [43] however the present study is the first to identify non-GAG-substituted forms of lumican in a specific spinal tissue. An alternative possibility is that the glucosamine-6-sulfate stub epitope identified by the BKS-1 antibody is absent in some forms of lumican or access to this epitope is sterically blocked by some other group on the GAG side chain and is thus non-reactive. Non KS substituted forms of fibromodulin have been described in canine articular cartilage [44]. Non-glycanated forms of decorin and biglycan in dentine are apparently due to proteolytic removal of their N-terminal GAG substituted regions [45]. In lumican, KS chains are distributed at a number of sites along the core protein and are not localized at the N-terminus like in decorin and biglycan [46]. GlcNAc 6-O-sulphotransferase (Chst5) knock-out mice have been developed to ascertain the role of 6-sulphation of N-acetyl glucosamine in KS on corneal development [47]. Lumican, the major corneal KS-PG was present as a GAG-free core protein form in the Chst5 null mouse with the corneal stroma displaying widespread structural alterations in collagen fibrillar architecture, decreased interfibrillar spacing, and general disorganization of the normal regular orthogonal arrangements of collagen fibers which are essential for corneal optical clarity [48]. The enzymatic sulfation of KS GAG chains is thus a key requirement in tissue morphogenesis and collagen matrix organization [47,49]. Not only is the directive role of the ECM now well appreciated in SC development [50] but its contributions to SC homeostasis in health and disease are also recognized [51] Furthermore, lumican is also an inhibitor of matrix metalloprotease (MMP)-14 and thus may regulate MMP-mediated remodeling and development of the SC ECM [52,53]. Lumcorin, a peptide derived from leucine rich repeat 9 of lumican, has cell regulatory roles via interactive properties with α2β1 integrin controlling cell migration and has angiostatic properties through its ability to inhibit MMP-9 and 14 and block α2β1 integrin interactions utilized by endothelial cells for tube formation [52,54,55,56]. Lumican is also a matrikine, [57,58] promoting wound healing and tissue homeostasis by modulating physiological and pathological gene expression [57,58,59,60,61]. A synthetic LumC13-terminal peptide [62] YEALRVANEVTLN (Lumikine) binds ALK5/TGFBR1 (type1 TGFβ receptor) through which it promotes wound healing [61,62]. Figure 7 outlines these features in a schematic showing the structural organization of lumican and illustrates potential roles for lumican in spinal development.

Keratocan also has neuro-directory, growth factor, and morphogen interactivities supportive of SC developmental processes [5,16,63]. The KS chains of keratocan have interactive properties with a range of neuroregulatory proteins which direct nerve migration in neural tissues [3,16]. Keratocan mRNA is developmentally controlled in the anterior–posterior and dorsal ventral axes during early (E2–E3) chick embryonic development [64]. Expression of keratocan in the lateral mesenchyme is accompanied by neural cell differentiation and extension ventrally in the CNS. In the developmental cornea, accumulation of highly sulfated PGs in the posterior stroma inhibits nerve penetration; however, keratocan in the anterior epithelium is more permissive of nerve penetration [65]. Trigeminal nerve growth in the embryonic chick reaches the corneal margin by E5, and is initially repelled by highly sulfated ECM PGs encircling the corneal margins over E5–E7 entering the cornea on E7 as illustrated in Figure 7a. KS mediated Robo-Slit cell signaling also guides trigeminal nerve development and migration [66]. KS has previously been a rather neglected GAG due to an absence of known interactive ligands relevant to tissue development. However, a number of KS-interactive morphogens, growth factors, and neuroregulatory proteins have now been identified [16] reinforcing the potential importance of keratocan and lumican in spinal development. Macrophages, reactive microglia, and oligodendrocyte progenitors but not astrocytes synthesize KS-PGs following SC injury in adult Fischer 344 rats [67]. KS interacts with synaptotagmin-2 like Rho GTPase activating protein 17 (ARHGAP17) [68] and synaptotagmin a synaptic vesicle membrane protein which co-ordinates neurotransmitter release [69,70]. ARHGAP17, a Rho GTPase-activating protein for Rac1 also regulates CDC42 activity and remodeling of the neuronal cytoskeleton critically maintaining cellular tight junctions [71,72], formation of complexin neuronal SNARE (soluble N-ethylmaleimide sensitive factor attachment protein receptor) complexes, and the coordination of the synchronous release of synaptic neurotransmitters during neuronal activation [73]. ARHGAP17 and synaptotagmin are two examples of KS-PG interactive regulators of Rho GTPase activation and cell signaling [71,72,73,74]. Slit and Robo also interact with KS to modulate axonal development critically regulating axonal outgrowth and directional extension [75]. KS is a unique extracellular matrix (ECM) component of the roof plate which acts as a barrier to axonal development directing axonal growth in the dorsal midline [76], inhibits commissural axonal elongation through the roof plate in the embryonic SC, and provides crucial directional cues to commissural axonal development and cortical interconnections between the two brain hemispheres ensuring effective communication between the hemispheres and co-ordination of motor responses, controlling gait, balance, and posture. 

In the present study, Western blotting with the KER-1 and LUM-1 antibodies alongside commercial keratocan and lumican antibodies validated both KER-1 and LUM-1 and confirmed that they detect 37 kDa keratocan and 51 kDa lumican core proteins in rat spinal tissues and human articular cartilage. BKS-1(+) also identified these core protein species. Higher power images of BKS-1(+) immunolocalizations in 4 month rat SC highlighted the subtlety in expression of KS on different cell types: BKS-1(+) identified positively-stained microglia throughout the grey and white matter whereas positively stained neurons were confined to the grey matter. The midline of the developing brain and SC is a place where axonal guidance decisions effects the patterning of sensory and motor systems. A variety of glial structures develop along the midline crucially directing axons to the left or right side of the CNS during spinal development [77,78,79]. KS has a diverse repertoire of interactive neuroregulatory ligands [1,3,16], and it remains to be determined how lumican and keratocan utilize these to direct neuroregulatory processes [1,3] in SC development. Moreover, binding of KS to *Shh* acts as a differential regulatory switch in the transformation of motor neurons to oligodendrocytes during SC development and re-myelination during axonal repair [80] supporting novel roles for keratocan and lumican in such processes. It is envisaged that future discovery of lumican and keratocan ligands will serve to further reinforce their roles in neural and skeletal development.

## 5. Conclusions

BKS-1 immunolocalizations are accurate quantitative measures of KS since one BKS-1 epitope is labeled per KS chain in all KS glycoforms irrespective of their charge status whereas MAb 5-D-4 binding is multivalent and identifies highly charged hexasaccharide KS epitopes. While this improves the sensitivity of detection of KS by MAb 5-D-4, it imparts a bias towards detection of highly sulfated KS chains. BKS-1 is therefore a useful new KS antibody with significant utility in identifying KS in early tissue development in KS-PGs containing low sulfation KS glycoforms as well as highly sulfated KS in mature tissues. During fetal development, BKS-1(+) detects a yet to be identified KS-substituted PG other than keratocan and lumican within the pre-ossification centers of the vertebral bodies. MAb BKS-1 also strongly delineates KS-PGs within the inner annulus of the developing IVD. In the adult rat, BKS-1(+) expression patterns correlate closely with KS SLRPs in the SC and IVD tissue. In fetal SC and vertebral spinal tissues a close correlation is observed between BKS-1(+) and lumican, but not keratocan expression which is absent from the IVD and only weakly present in GP. The vertebral growth plate of the rat spine also appears to contain a non-glycanated form of lumican which awaits further characterization.
